# Characterization of *Lysobacter enzymogenes* B25, a potential biological control agent of plant-parasitic nematodes, and its mode of action

**DOI:** 10.3934/microbiol.2023010

**Published:** 2023-03-01

**Authors:** Sònia Martínez-Servat, Lola Pinyol-Escala, Oriol Daura-Pich, Marta Almazán, Iker Hernández, Belén López-García, Carolina Fernández

**Affiliations:** Futureco Bioscience, S.A, Olèrdola, Barcelona, Spain

**Keywords:** secondary metabolites, HSAF, twitching motility, lytic enzymes, induction of host plant immunity, nematicidal activity, *Meloidogyne*, biocontrol

## Abstract

It is certainly difficult to estimate productivity losses due to the action of phytopathogenic nematodes but it might be about 12 % of world agricultural production. Although there are numerous tools to reduce the effect of these nematodes, there is growing concern about their environmental impact. *Lysobacter enzymogenes* B25 is an effective biological control agent against plant-parasitic nematodes, showing control over root-knot nematodes (RKN) such as *Meloidogyne incognita* and *Meloidogyne javanica*. In this paper, the efficacy of B25 to control RKN infestation in tomato plants (*Solanum lycopersicum* cv. *Durinta*) is described. The bacterium was applied 4 times at an average of concentration around 10^8^ CFU/mL showing an efficacy of 50–95 % depending on the population and the pressure of the pathogen. Furthermore, the control activity of B25 was comparable to that of the reference chemical used. *L. enzymogenes* B25 is hereby characterized, and its mode of action studied, focusing on different mechanisms that include motility, the production of lytic enzymes and secondary metabolites and the induction of plant defenses. The presence of *M. incognita* increased the twitching motility of B25. In addition, cell-free supernatants obtained after growing B25, in both poor and rich media, showed efficacy in inhibiting RKN egg hatching *in vitro*. This nematicidal activity was sensitive to high temperatures, suggesting that it is mainly due to extracellular lytic enzymes. The secondary metabolites heat-stable antifungal factor and alteramide A/B were identified in the culture filtrate and their contribution to the nematicidal activity of B25 is discussed. This study points out *L. enzymogenes* B25 as a promising biocontrol microorganism against nematode infestation of plants and a good candidate to develop a sustainable nematicidal product.

## 1. Introduction

Nematodes are among the most widespread organisms on Earth. Of the about 25000 species that have been described [1], approximately 4300 (corresponding to 197 genera) have been reported as plant parasitic nematodes [2]. Their feeding process damages the root system of the plant, reducing its ability to absorb water and nutrients. In the presence of nematodes, symptoms such as galls or necrotic lesions are observed. These alterations in the tissues often lead to chlorosis, growth retardation, wilting and dwarfism, which globally translates into weak-growing plants, which ultimately affects crop productivity.

It is certainly difficult to estimate productivity losses due to the action of nematodes but various authors mention that it might be about 12% of world agricultural production [3]. This estimate is an average that varies greatly depending on the crop. For potatoes, losses have been estimated worldwide between 10–15% of the harvest, representing 78 billion USD [4] whereas losses on rice yield due to *Meloidogyne graminicola* range from 20–80% [5]. Another aspect that must be taken into account is that frequently, the damage caused by nematodes is confused with fungal or bacterial attacks, water stress, or other physiological imbalances [6]. Therefore, it is very possible that yield losses are also being underestimated.

There are many tools to reduce the effect of nematodes such as applying crop rotations, biofumigation, cover crops, fallow, trap crops, soil amendments, soil solarization, and authorized chemical, botanical and microbial pesticides [7]. Regarding the last, it is worth noting that there are very few microorganisms registered for use as nematicides worldwide. To our knowledge, only four nematicidal strains are registered in Europe (https://ec.europa.eu/food/plant/pesticides/eu-pesticides-database/active-substances/?event=search.as; https://www.mapa.gob.es/es/agricultura/temas/sanidad-vegetal/productos-fitosanitarios/registro-productos/), namely *Bacillus firmus* I-1582, *Paecilomyces lilacinus* 251, *P. lilacinus* Pl11, and *Pasteuria nishizawae* Pn1.

Numerous reports have described the biocontrol activity of the genus *Lysobacter*, in particular *L. enzymogenes*, *L. capsicis* and *L. antibioticus*, against plant diseases caused by bacteria, fungi, oomycetes and nematodes and their mode of action has been extensively studied [8]–[12]. Research on *L. enzymogenes* as biocontrol agent of plant-parasitic nematodes is very limited in comparison to the numerous reports relating to its activity against fungal and oomycetous pathogens. Chen and coworkers reported that *L. enzymogenes* C3 was able to lyse several plant-parasitic nematode species *in vitro*, including *M. javanica*, *Heterodera schachtii*, *Pratylenchus penetrans* and *Aphelenchides fragariae* [13]. *Lysobacter enzymogenes* C3 culture filtrate also reduced the survival of juveniles suggesting that the bacterium is able to produce and secrete some nematicidal compounds. In nematodes, chitin occurs as the second layer of the eggshells, so the production of chitinase by *L. enzymogenes* could be the mechanism for a decrease in egg viability. However, chitinolysis cannot explain the rapid inactivation of vermiform nematodes *P. penetrans* and *A. fragariae* suggesting that other enzymes or metabolites secreted by *L. enzymogenes* might be responsible for the effect of the bacterium over nematodes. Recently, the nematicidal activity of *L. enzymogenes* C3 against different life stages of *Heterodera glycines* and *H. schachtii* has been reported under growth chamber conditions [14]. Yuen and coworkers described that nematodes exposed to mutant strains of *L. enzymogenes* blocked in the production of heat-stable antifungal factor (HSAF) survived and reproduced, demonstrating that HSAF production is an important mechanism involved in the biocontrol activity of *L. enzymogenes* against cyst nematodes [14].

*Lysobacter* members, like other bacteria lacking flagella, are able to move by the presence of thin and long filamentous appendages on the surface of bacteria called type IV pili (T4P). These are involved in, biofilm formation, interbacterial binding, DNA uptake, phage transduction, pathogenic activity and twitching motility [15]. Twitching is a highly organized multicellular collective behavior that is considered essential for bacterial propagation and colonization within host xylem vessels [10]. T4P-mediated twitching is critical for *L. enzymogenes* to move vigorously in its natural ecosystem (e.g., soil and plant surface) and it is associated with predatory behavior towards other microorganisms [17].

In addition to its ability to move in the environment, several secondary metabolites and enzymes are involved in the antimicrobial mode of action of *L. enzymogenes*. On the one hand, it has been widely reported that the polycyclic tetramate macrolactam HSAF has an important role to control plant disease caused by phytopathogenic fungi [18],[19]. The target of HSAF is the enzyme ceramide synthase implicated in the biosynthesis of sphingolipids and required for filamentous fungi to grow [20]. On the other hand, the main metabolite conferring *L. enzymogenes* antibacterial activity is the cyclic depsipeptide WAP-8294A2, which selectively recognizes menaquinone present in the cytoplasmic membrane of gram-positive bacteria leading to the disruption and lysis of the membrane [21]. Moreover, *L. enzymogenes* secretes numerous extracellular lytic enzymes, such as chitinases, proteases, and lipases, which are able to degrade the major cell wall components of various phytopathogenic fungi, oomycetes, bacteria or nematodes, causing their death [17],[22],[23].

The *clp* gene product is a master regulator of T4P-dependent twitching and the production of lytic enzymes and antifungal metabolites in *L. enzymogenes* [16],[24]. However, the treatment of plants with a *clp*^-^ mutant strain still resulted in a slight biocontrol of summer patch disease caused by *Magnaporthe poae* [8]. Therefore, additional mechanisms not controlled by the *clp* gene may be operating. In fact, plant-mediated traits such as induced resistance, growth stimulations, and enhanced nutrient availability could be linked to the biological control activity of *L. enzymogenes* [25],[26].

In this study, the biocontrol efficacy of B25 on root-knot nematodes in tomato plants (*Solanum lycopersicum* cv. Durinta) under climatic chamber conditions has been investigated. Moreover, the purpose of this study has been to describe the role of lytic enzymes and secondary metabolites, motility, and induction of plant defenses on the mode of action of B25 as a nematicide.

## 2. Materials and methods

### 2.1. Strain isolation and identification

*Lysobacter enzymogenes* B25 was isolated from a pepper plant harvested from an agricultural field managed according to organic agriculture practices located in Gavà (northeast Spain). Identification at the species level and genome sequencing analysis was previously performed [27].

### 2.2. Effect of temperature, pH and salinity on growth rate

Bacterial growth was measured at different temperatures (4 °C, 18 °C, 22 °C, 25 °C, 28 °C, 30 °C, 34 °C, 37 °C, and 40 °C) and pH range between 3–12 at intervals of 1 pH unit, using nutrient agar (NA) medium as a substrate. The ability of B25 to grow in different salt concentrations was carried out by inoculating bacterial culture in sterile 96-well plates. For the salt tolerance experiment, B25 was cultured overnight in Luria-Bertani (LB) liquid medium (5 g/L of yeast extract, 10 g/L of tryptone and 10 g/L of NaCl) with continuous shaking at 28 °C and 200 rpm. From this stationary culture, 100 µL of bacterial suspension with a final optical density (OD) at 600 nm of 0.01 were added to the wells containing the following final concentrations of NaCl (%): 0, 0.5, 1, 2, 3, 4, 5, 6, 7, 8, 9, and 10. The plate was read after 48 h of incubation at 28 °C using a microplate reader (Multilabel Plater Reader HALO LED96, Dynamica).

### 2.3. Cell motility: swarming and twitching assays

Swarming motility assays were performed according to the methodology previously described by other authors [28]. In brief, swarming assays were performed in two different media: modified M9 salts medium without NH_4_Cl (0.5 % Casamino Acids, 2 mM MgSO_4_, 0.1 mM CaCl_2_) supplemented with 0.4 % glucose and BM2 medium (0.5 % Casamino Acids, 62 mM KH_2_PO_4_, 1 M MgSO_4_, 10 mM FeSO_4_, 0.4 % glucose). Both media were solidified with 0.5% Noble agar. Plates containing 20 mL of fresh swarm medium were dried under a laminar-flow hood for 20 min before inoculation. Bacteria were inoculated by picking fresh colonies using a Drigralski spatula and depositing it on the top of the swarm plate. Plates were sealed to maintain the humidity and incubated overnight at 28 °C or 37 °C. Pictures were taken from a representative plate out of three independent experiments.

Twitching motility was assayed by two methods. (I) Macroscopic twitching assays were evaluated on TSA 1/20 plates [0.15 % TSB broth (17 g/L tryptone, 3 g/L soy peptone, 5 g/L NaCl, 2.5 g/L K_2_HPO_4_, 2.5 g/L glucose) + 1 % (w/v) agar] as previously described [29], with some modifications. Twitch plates were briefly dried and B25 was stab inoculated with a needle to the bottom of the Petri dish from an overnight-grown NA plate. Plates were incubated at 28 °C for 72 h. To aid visualization, the zone of interstitial biofilm expansion at the agar and petri dish interface was visualized by flooding the plate with a TM developer solution (50 % methanol and 10 % acetic acid) or with crystal violet [1 % (wt/vol) solution] after removing the agar layer and washing unattached cells with water, as described [30]. (II) Microscopic twitching assays were carried out essentially as previously described [10],[17],[29]. A folded 9 × 9 cm piece of sterile filter paper was placed on a side of a dish. Then, a sterile glass slide was placed next to the filter paper, and 2 mL of sterile distilled water was added to the paper to provide a moist environment. Subsequently, 1 mL of twitching medium (0.8 % agar, 0.4 % tryptone, 0.2 % yeast extract, 0.2 % NaCl, 0.1 % MgSO_4_·7H_2_O) was evenly distributed on the slide and let solidify for 15 minutes. With a plastic loop, from the outer edge of a fresh bacterial colony, a surface in 1 mm of diameter was inoculated to the slide. Besides, a sterile coverslip was placed over the inoculation point. Then, 20 µL of *M. incognita* suspension, at 1500 J2/mL concentration, was dropped next to the coverslip. Control samples did not contain nematode suspension. After 24 h at 28 °C of incubation, the margin of bacterial culture was observed under a microscope (MOTIC BA310) with 400-fold magnification. Twitching motility was observed when bacterial cells emerged from the edge of the bacterial colony. Three replicates for each treatment were used, and each experiment was performed three times.

### 2.4. Biofilm formation in microtiter plates and glass test tubes

Quantification of biofilm formation in sterile untreated 96-well microtiter plates was performed by crystal violet staining, as previously described [28]. In summary, overnight culture was grown in LB at 28 °C with 200 rpm shaking, followed by dilution of the culture into fresh LB to an OD_550_ of 0.1. A volume of the bacterial suspension (200 µL) was inoculated into the wells of microtiter plates and incubated for 48 h at 28 °C. Prior to biofilm quantification, total cell biomass was estimated by measuring the OD_620_ using a microplate reader (Multilabel Plater Reader HALO LED96, Dynamica). Then, wells containing adhered cells were washed three times with water, fixed at 60 °C for 1 h, and stained for 15 min with 200 µL of 0.1 % crystal violet. The stained biofilms were rinsed with distilled water and allowed to dry for 30 min at 37 °C. Bound crystal violet was extracted from the stained biofilm with 95 % ethanol and the absorption was photometrically measured at 550 nm (OD_550_) using a plate reader. Biofilm formation (OD_550_ of crystal violet) was normalized by cell growth (OD_620_) and reported as the relative biofilm formation. For this quantitative assay, sixteen replicate wells were done. In addition, biofilm formation on the glass surface was assayed by inoculating 3 mL of the same bacterial suspension into glass test tubes. After incubation at 28 °C and 250 rpm for 48 h, biofilm formation was measured by crystal violet staining as described above.

### 2.5. Enzymatic activity assays

Enzymatic activities from cells and cell-free culture supernatant of B25 were determined using API-ZYM^®^ (bioMérieux, Marcy l'Etoile, France), according to the manufacturer's instructions. The supernatant was obtained from a B25 fermentation in the following conditions: VEG medium (10 g/L yeast extract, 10 g/L glucose, 0.1 g/L KH_2_PO_4_, 0.1 g/L K_2_HPO_4_ and 0.2 g/L MgSO_4_ × 7H_2_O), 30 °C and 24 h. After fermentation, the culture was centrifuged at 5000 g for 10 min (Hitachi CR22N) and the supernatant was filtered by passing through a 0.22 µm pore size polyethersulfone (PES) filter to obtain the cell-free culture supernatant. To confirm that the supernatants were free of bacterial cells, aliquots of 100 µL were plated in NA and the absence of growth was confirmed after incubation at 28 °C for 2 days.

In addition, the presence of chitinase, protease, lipase, cellulase, and gelatinase activity were analyzed using classic methods. Briefly, 10 µL of overnight grown culture were spot plated on chitin agar medium [31], casein agar medium [32], tween 80 agar medium [33] and microcrystalline cellulose (MC)-agar medium [34]. Plates were incubated for seven days at 28 °C. For observation, the cellulase detection plates were stained using 1 % Congo red dye and, after 15 min, the stained plates were rinsed with 1 M NaCl solution. In all cases, the appearance of a clear halo around colonies indicates enzymatic degradation. On the other hand, the qualitative assay for gelatinase activity was determined by using the nutrient gelatin plate method and nutrient gelatin stab method, as described [35].

### 2.6. Biosurfactant activity

The surfactant activity or the ability to collapse a droplet of water was tested as described [36]. Twenty µL of cell-free culture supernatants were pipetted as a droplet onto parafilm. Subsequently, 3 µL of the Congo red dye (which had no influence on the shape of the droplets) was added to stain the water and supernatants for photographic purposes. The spreading of the droplet on the parafilm surface was followed over hours until the droplet dried. The diameter of the dried droplet was recorded.

### 2.7. Efficacy assessment on root-knot nematodes in tomato plants under growth chamber conditions

Tomato (*Solanum lycopersicum*) cv. Durinta seeds were surface-sterilized, sown in a sterile substrate (vermiculite), and grown in a growth chamber (Fitoclima D-1200^®^, Aralab) set at 24/20 °C (day/night), 65 % relative humidity, and a photoperiod of 16/8 h (day/night). After 28 days, the plants were transplanted into 3-L pots filled with a commercial substrate (8–14 % organic matter, pH 7.0–8.0, 50–120 mS/m, 95 % particles < 12 mm diameter, mineral fertilization 20-10-15 NPK + 2 Mg; Burés Professional S.A., reference B32002). Three bioassays under growth chamber conditions are described in this study. In the first bioassay, the substrate was artificially infested with a mixed population of *M. incognita* and *M. javanica* isolated from infested tomato plants from a commercial tomato field in Viladecans, Spain, at a rate of 6000 eggs per pot. In bioassays 2 and 3, the plants were inoculated with *M. javanica* isolated from infested tomato plants from a commercial tomato field in Almería, Spain, at a rate of 10000 eggs per pot. B25 was applied 4 times. The first application was done immediately after the plants were transplanted [0 days after transplant (0-DAT)], and then at 7, 21, and 35 DAT. In the three bioassays, the plants were inoculated with nematodes 24 h after the transplant, so the first application of B25 was before inoculation with nematodes. The applications consisted of 50 mL (bioassay 1) or 30 mL (bioassays 2 and 3) of B25 per plant (aqueous suspension), at an average concentration of 10^8^ CFU/mL (bioassay 1) and 2 × 10^8^ CFU/mL (bioassays 2 and 3). In all three bioassays, the reference chemical Sondae^®^ (Oxamyl 10 % w/v) was applied 3 times in 14-day intervals, starting 7 days after transplant.

Sixty days after transplant the number of nematode eggs per g of root (EgR) and the number of eggs per plant (EP) were determined in the tomato plant roots. For this, the roots were gently rinsed with tap water, ground in a household blender with a solution of 10 % bleach, passed through 100, 75 and 25 µm meshes and the eggs were counted in a McMaster counting chamber under the light microscope.

After verifying the homoscedasticity and normality of the counting data, ANOVA and LSD post hoc tests were performed. Differences were considered statistically significant at P < 0.05. Statistical analyses were determined with R.

### 2.8. Efficacy assessment on root-knot nematodes in tomato plants under greenhouse conditions

B25 has been tested in several greenhouse trials in tomatoes artificially infested with nematodes (with *M. incognita* plus *M. javanica* or with *Meloidogyne hapla*). The substrate was inoculated with 6000 eggs per pot 24 h after the transplant. B25 was applied 4 times (at 0, 7, 21 and 35 DAT) at a rate of 50 mL per plant. In each bioassay a reference chemical and/or biological was used. The reference chemical Oxamyl 10 % was applied 3 times in 14-day intervals, starting 7 days after transplant; the reference chemical Flupyram 40% was applied twice in 21-day intervals; and the biological reference *Bacillus firmus* was applied as B25.

Greenhouse conditions depended on the season. In winter (assays 181211 and 190122 in Table S1) the maximum and minimum temperatures were set at 24 °C and 18 °C, respectively. On the other hand, in summer no temperature was set.

### 2.9. High-performance liquid chromatography (HPLC) analysis for metabolite production

Considering the conditions previously published for metabolite production [18],[37], B25 was grown in VEG medium (as described above) or 1/10-strength tryptic soy broth (TSB 1/10: 1.7 g/L tryptone, 0.3 g/L soy peptone, 0.25 g/L glucose, 0.5 g/L NaCl and 0.25 g/L K_2_HPO_4_).

Cell-free culture supernatants were analyzed by HPLC (2696 Alliance equip with 2998 photodiode array detector, Waters). Chromatography was performed using an XBridge C18 reversed-phase column (5 µm 4.6 × 150 mm, Waters) eluted at 1 mL/min of mobile phase, which consisted of a mix of solvents A [water + 0.05 % trifluoroacetic acid (TFA)] and B (acetonitrile + 0.05 % TFA). The linear gradient was 0–10 % B for 10 min; 10–40 % B for 3 min; 40–100 % B for 3 min; 0 % B for 8 min. Chromatograms were obtained by processing at 254 nm and compared with the culture medium to identify specific compounds produced by this bacterium.

Cell-free culture supernatant of B25 was submitted to HPLC-high-resolution mass spectrometry (HRMS) analysis (Separative Techniques Unit, Scientific and Technological Center, University of Barcelona, Spain). The liquid chromatography was performed using an Accela chromatograph (Thermo Instruments) with a Zorbax Eclipse XDB-C18 column (5 µm 4.6 × 150 mm) as the stationary phase. The gradient was the same as described above but with the solvents A (water + 0.2 % CH_3_COOH) and B (acetonitrile + 0.2 % CH_3_COOH). For mass spectrometry analysis, the equipment was an LTQ Orbitrap Velos (Thermo Instruments). The capillary temperature was 375 °C, source voltage 3.5 kV, acquisition FTMS (+), and full scan of m/z 100–1000.

### 2.10. Metabolite purification by preparative HPLC

Metabolites produced by *L. enzymogenes* B25 were purified from cell-free supernatant by preparative HPLC (Delta Prep 4000, Waters) with a 432 detector (Konron Instruments). The stationary phase was an OBD SunFire PrepC reversed-phase column (10 µm 19 × 250 mm, Waters). The mobile phase consisted of a mix of solvents A [water + 0.05 % trifluoracetic acid (TFA)] and B (acetonitrile + 0.05 % TFA). The linear gradient was 50 % B for 2 min; 50–70 % B for 8 min; 50 % B for 3 min. The flow was 15 mL/min.

The fractions of interest were manually collected and concentrated by a rotatory evaporator. The identity of the purified metabolites was confirmed by Electrospray ionization time-of-flight (ESI-TOF) mass spectrometry analysis (Scientific and Technological Centers, UB, Barcelona, Spain) using a liquid chromatography equipment (model G1969A, Agilent Technologies) with a dual-nebulizer electrospray ion source.

### 2.11. In vitro nematicidal activity of metabolic fractions

An assay was designed to measure the egg-hatching rate of a root-knot nematode population in the presence of different metabolic fractions. These fractions were cell-free culture supernatant of B25 after growing in two different media (TSB 1/10 and VEG). In addition, both fractions were subjected to a heat treatment process that consisted of heating at 80 °C for 15 min.

The original root-knot nematode population, composed of a mixture of *M. incognita* and *M. javanica* at unknown proportions, was isolated from a tomato field in Viladecans (Spain). This population was maintained in the roots of susceptible tomato plants (cv. Marmande Cuarenteno) grown in a greenhouse. Sand-loaded sieves were disposed on multiwell plates used as hatching chambers (Nunclon™ Surface multiwall plates, Nunc, Denmark). The sieves were pre-soaked with sterile distilled water, inoculated with a freshly isolated nematode egg suspension (around 500 eggs per sieve) and then treated with 200 µL of ¼ dilution of each metabolite sample.

The hatching chambers were covered with a lid and incubated at 26 °C for 2 weeks. The egg hatching rate was calculated (number of juveniles recovered/nº of eggs inoculated x 100) at 7 and 14 days after inoculation. Each treatment was replicated 4 times including a negative control with a chemical reference product at the commercially recommended dose and a control with sterile distilled water (to establish a reference to compare the results to the normal hatching percentage).

### 2.12. Induction of plant immune responses

Tomato seeds (cv. Marmande Cuarenteno) were surface-sterilized and grown as described before. Twenty-eight day-old plants were transplanted to 3-L pots and transferred to a greenhouse. Starting the day of transplantation, B25 was applied 4 times in 7-day intervals by delivering 30 mL of a B25 solution, containing a total of 5 × 10^7^ CFU for each application, directly to the substrate at the base of the shoot. Seven days after the last application, the terminal leaflet of the youngest fully developed leaf was collected from each plant. The leaflets of 4 replicate plants were pooled and immediately frozen in dry ice. The samples were kept at −80 °C until analysis.

Total RNA was extracted from the samples using Ribozl according to the manufacturer's instructions (Amresco, Solon, OH). The concentration and purity of the total RNA was determined by microvolume spectrophotometry (DS-11 FX, Denovix). The total RNA (7 µg) was digested with DNaseI according to the manufacturer's instructions (Sigma-Aldrich). The resulting DNA-free RNA (6 µg) was used as a template to synthesize first-strand cDNA by reverse transcription using High Retrotranscriptase according to the manufacturer's instructions (Biotools, Spain) and oligo dT_18_ primer. The cDNA was diluted to 30 ng/µL for real-time quantitative PCR (qPCR).

The relative expression levels of plant immune response marker genes (Table S2) were measured by qPCR with intercalating fluorophore chemistry (SYBR Green I, Roche Life Science) in biological triplicates, according to the master mix manufacturer's instructions, and applying the 2^-ΔΔCt^ method for quantifications [38]. All primer pairs used in the present study were previously reported in the literature as markers of plant defense responses in tomatoes [39]–[44] (see Tables S2 and S3 for details on primers and raw data, respectively). Pathogenesis-related protein 1 (PR1), is a multi-gene family of secreted proteins with an unclear function that has been broadly reported to be upregulated during defense responses [45],[46]. Similarly, PR2's and PR3's are families of β-1,3-glucanases and chitinases, respectively, broadly reported to be involved in the degradation of the pathogen cell wall or cuticle components and to experience an increment in their expression levels upon treatment with jasmonate, ethylene and/or pathogen challenge (see, for instance, [47],[48]) (for cross-reference, the *PR1*, *PR2* and *PR3* genes analyzed hereby correspond to Solyc09g007010.1, Solyc01g008620.2 and Solyc10g055810.2, respectively). RCR3 is a papain-like cysteine protease responding to pathogen infection [41]. PINII is a protease inhibitor responding to wounding, jasmonic acid and arbuscular mycorrhiza [39]. And 4CL (4-coumarte:CoA ligase) is a key enzyme in the biosynthesis of complex phenylpropanoids, many of which display a phytoalexin role. Although little is known about the regulation of 4CL in tomatoes, this gene has been reported to respond to wounding, pathogen challenge, stress hormones, etc. in closely related species [49]–[52]. EF1α was used as a reference gene because in a previous study, we determined that the expression of this gene was more stable than that of UBI3 [39] and RCR3 [42] (the standard deviation of the Ct's were 1.37, 1.44 and 1.92, respectively; n = 15) in tomato plants treated with salicylic acid (400 µM), pyocyanin (10 µM), and other treatments modifying plant immunity.

## 3. Results

### 3.1. Phenotypic characterization

*Lysobacter enzymogenes* B25 is a Gram-negative and bacillus-shaped non-flagellated bacterium, 1–1.7 µm long. B25 can grow at pH 4 to 10, 18 to 37 °C and 0 to 2 % NaCl (Table 1). Consequently, B25 is considered a neutrophilic, mesophilic and non-halotolerant strain.

**Table 1.** Phenotypic characterization of B25.

Morphological characteristics		

Shape	bacillus (rod-shape)
Size (µm)	1–1.7
Gram-staining	negative
Cell motility	Swarming positive
	Twitching positive

Growth conditions
Range of growth temperature (optimal)	18 °C to 37 °C (28 °C to 35 °C)
Range of growth pH (optimal)	4 to 10 (7 to 9)
Tolerance to NaCl (optimal)	0 to 2 % (0 to 0.5 %)
Physiology and metabolism

Enzymes	API-ZYM^®^	Cells: Alkaline phosphatase, Esterase Lipase (C 8), Lipase (C 14), Leucine arylamidase, Valine arylamidase, Trypsin, Acid phosphatase, Naphtol-AS-BI-phosphohydrolase, N-acetyl-β-glucosaminidase
		Supernatant: Alkaline phosphatase, Esterase (C 4), Esterase Lipase (C 8), Leucine aryamidase, Valine arylamidase, α-chymotrypsin, Acid phosphatase, Naftol-AS-BI-phosphohydrolase
	Chitinase	Positive
	Protease	Positive
	Lipase	Positive
	Cellulase	Positive
	Gelatinase	Positive
Biofilm formation	Positive

**Figure 1.**
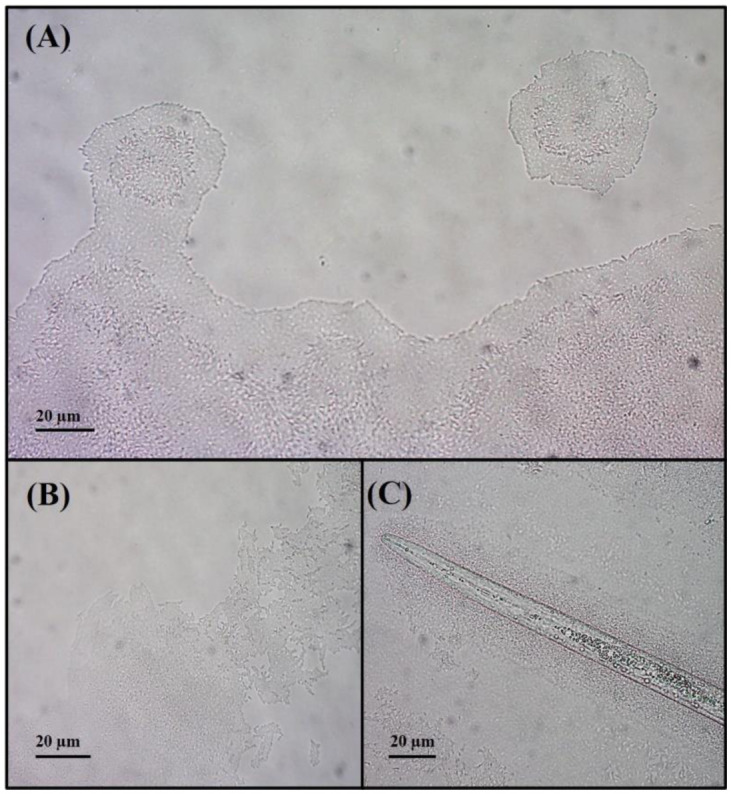
*Lysobacter enzymogenes* B25 Microscopic twitching (magnification x400). (A–B) B25 twitching modalities. (A) Leading edge raft (LER) formation. (B) Disaggregation of principal colony margin with non-uniform cell migration. (C) High-density cell envelope around the *M. incognita* formed by B25.

**Figure 2.**
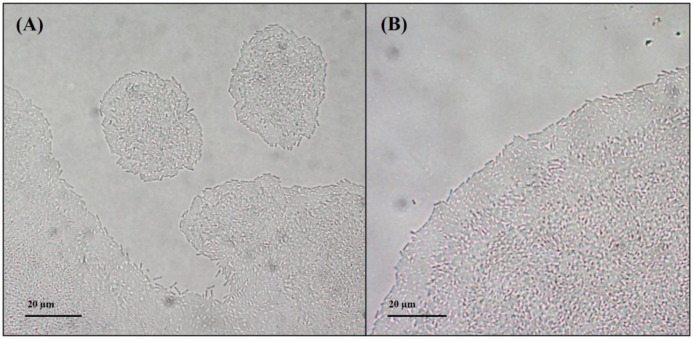
Twitching motility of *Lysobacter enzymogenes* B25 in the presence or absence of *Meloidogyne incognita* (magnification x400). 62% of samples containing *M. incognita* (A) increased B25 twitching motility and disaggregation of colony edges, compared to control samples (B) which presented mainly uniform and conserved margins.

Biofilm formation was evaluated on a polystyrene or glass surface. The results showed that B25 has the capacity to form biofilm on both surfaces (Figure S1). In addition, B25 was able to swarm in different conditions (Figure S2) and exhibited surface twitching motility (Figures 1 and 2). Specifically, macroscopic twitching-motility assays revealed that B25 formed a small twitch zone between the agar and the bottom of the petri dish (data not shown). In addition, light microscopy was used to examine the collective and individual behaviors of B25 that occur at the interstitial interface of a glass coverslip and a pad of nutrient media solidified with 0.8 % agar. Under these conditions, the colonies of B25 presented a high cell density in the inner portion of the colony, while a band of lower cell density was observed in the outer margins. B25 showed twitching motility, according to the leading edge raft (LER) modality [29]. These are large groups of cells emerging from the main margin of the colony, that migrate away from it. B25 LER maintains the same structure as the main margin: higher cell density in the center, and a band of lower cell density on the outer edge (Figure 1A). Apart from LER, B25 also presented another twitching modality, which consisted of the disaggregation of the principal colony margin with non-uniform cell migration (Figure 1B). B25 presented twitching in the absence or presence of *M. incognita*. However, 62 % of the samples containing *M. incognita* increased twitching motility compared to control samples (Figure 2). Furthermore, it was observed that B25 was capable of forming a high-density cell envelope around the nematode (Figure 1C and Figure S3). It is known that cell motility facilitates bacterial colonization on pathogen surfaces and the data of this study showed changes in the twitching motility of B25 when co-cultured with *M. incognita,* an important characteristic for the biological control activity of B25.

**Figure 3.**
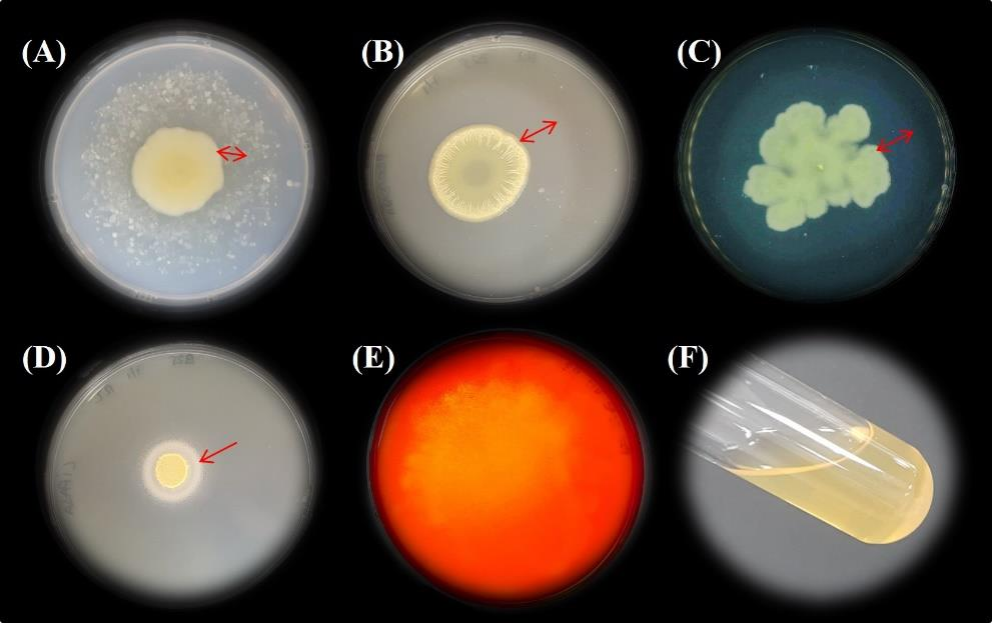
*Lysobacter enzymogenes* B25 grown on different enzyme determination media. (A) Chitin agar; red arrow indicates a clear zone around the colony due to chitinase production. (B) Casein agar; red arrow indicates a clear zone around the colony formed by protease/caseinase enzymes. (C) Nutrient gelatin plate; gelatin hydrolysis was indicated by the clear zone around the colony, indicated by the red arrow, after the addition of saturated ammonium sulfate. (D) Tween 80 opacity test; red arrow indicates the presence of white precipitation halo around the inoculation due to lipase/esterase activity. (E) Microcrystalline cellulose agar; cellulolytic enzymes formed a clear halo zone after being revealed with congo red 1 %. (F) B25 incubated in a nutrient gelatin tube exhibited positive gelatin hydrolysis, as shown by medium liquefaction maintenance, and no solidification, after 30 minutes in an ice bath.

According to API-ZYM^®^, B25 produces alkaline phosphatase, esterase (C4), esterase lipase (C8), lipase (C14), leucine arylamidase, valine arylamidase, trypsin, α-chymotrypsin, acid phosphatase, naphtol-AS-BI-phosphohydrolase, and N-acetyl-β-glucosaminidase (Table 1). Moreover, B25 secretes chitinase, protease, lipase, cellulase and gelatinase (Table 1). After incubation, enzymatic degradation was observed in all tested media due to the appearance of a clear halo around colonies and complete liquefaction was detected in the gelatin hydrolysis tubes (Table 1, Figure 3). These data suggest that the ability of B25 to produce abundant extracellular enzymes may be partly responsible for its antagonistic activity.

**Figure 4.**
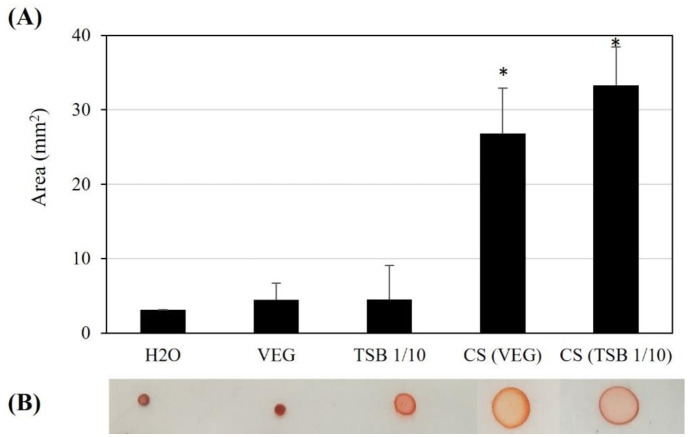
Biosurfactant activity of cell-free culture supernatant of *Lysobacter enzymogenes* B25 grown in VEG and TSB 1/10 media. (A) Area (mm^2^) obtained after drying 20 µL droplets of each cell-free culture supernatant (CS) onto parafilm. As controls, water and both culture media were used. Statistically significant differences of each CS sample with the corresponding control (T-test; p < 0.05) are indicated by “*”. (B) Representative images of each sample. Congo Red dye was added to stain the samples for photographic purposes.

To test whether *L. enzymogenes* B25 secretes compounds that decrease the surface tension of the culture media, a simple assay on parafilm was done with the samples from the cell-free culture supernatant (Figure 4). This strain produces a surfactant compound after growth on rich and poor media, VEG and TSB 1/10 respectively. Moreover, the surfactant activity was kept after heat treatment indicating that the surfactant compound is heat-stable (data not shown).

### 3.2. Nematicidal activity of B25

Firstly, the nematicidal activity of B25 on tomato plants (*Solanum lycopersicum* cv. *Durinta*) was tested in three bioassays under climatic chamber conditions. As described before, in the first bioassay the substrate was artificially infested with a mixed population of *M. incognita* and *M. javanica* but in the second and third tests, the nematode was *M. javanica*. The inoculation of the substrate with the initial inoculum was higher in bioassays 2 and 3 (10000 nematodes/plant) than in bioassay 1 (6000) due to the higher reproductive rate of the mixed population of *M. incognita* and *M. javanica* than the population *M. javanica*.

**Figure 5.**
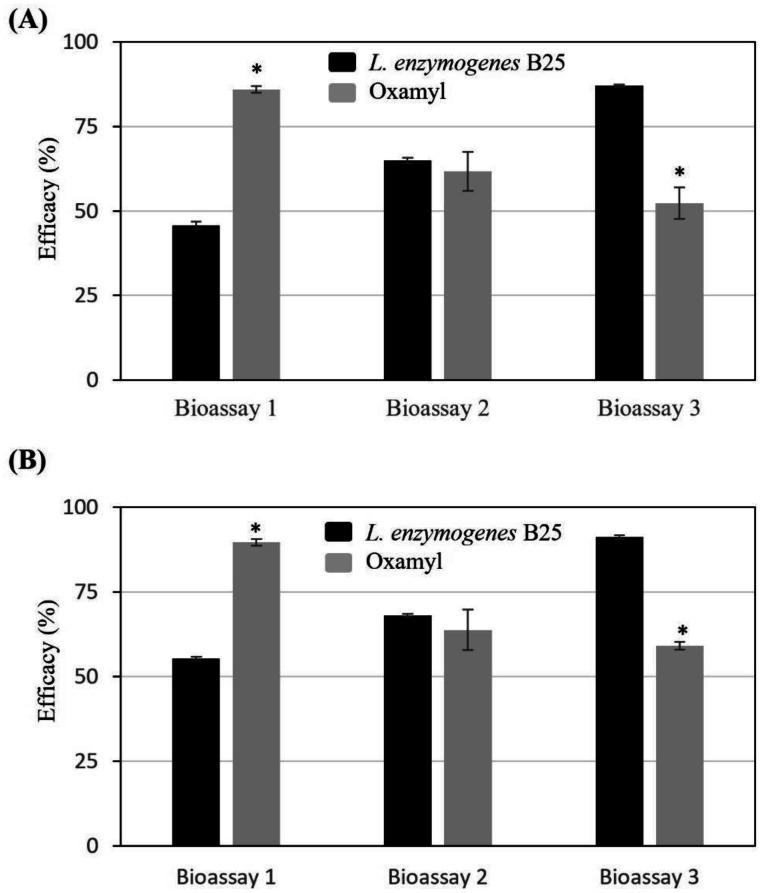
Nematicidal activity of *Lysobacter enzymogenes* B25 on tomato plants. Results are shown as percentage of efficacy by the different treatments regarding the eggs per g of root (A) and per plant (B) when compared to the chemical product (grey bars) (ANOVA and LSD post hoc; P < 0.05, ±SEM). Statistically different values are indicated by “*”.

In the first bioassay, the level of infestation at 60 days after the first application was considered high since untreated control plants presented an average of 25619 eggs/g of root and 429436 eggs/plant (Figure 5). In relation to the efficacy parameters evaluated, the plants treated with B25 reached efficiencies of 55.3 % concerning the number of eggs/plant (EP) and 45.8 % eggs/g of root (EgR), whereas the chemical standard (Oxamyl 10 % w/v) efficacies were 89.6 % and 86.0 %, respectively.

In a second bioassay with a moderate level of infestation (7126 eggs/g, equivalent to 144994 eggs/plant) compared with bioassay 1, there were no statistical differences between plants treated with B25 or the chemical standard, reaching efficiencies in the plants treated with B25 between 68.1 % (EgR) and 64.9 % (EP) according to the parameter analyzed. The efficacy observed in plants treated with the chemical product was 61.7 % (EgR) and 63.8 % (EP).

Finally, in the last trial also with a moderate level of infestation (7016 eggs/g, equivalent to 201149 eggs/plant), the performance of B25 was significantly better than that of the reference chemical. The plants treated with B25 reached efficiencies of 91.1 % (EgR) and 87.0 % (EP), whereas the ones treated with oxamyl, efficacies were between 59.2 % (EgR) and 52.3 % (EP).

In addition, the efficacy of strain B25 has been demonstrated in several greenhouse trials in tomato artificially infested with a mixed population of *M. incognita* and *M. javanica* or with *M. hapla* (Table S1). Moreover, Good Experimental Practice (*GEP)* field trials assessing B25 against RKN in tomato, cucumber, carrot and melon plants, and against cysts of *Globodera pallida* and *Globodera rostochiensis* in potato plants have been performed by different Contract Research Organizations (CROs) (data not shown).

### 3.3. Identification of potential nematicidal metabolites produced by B25

**Figure 6.**
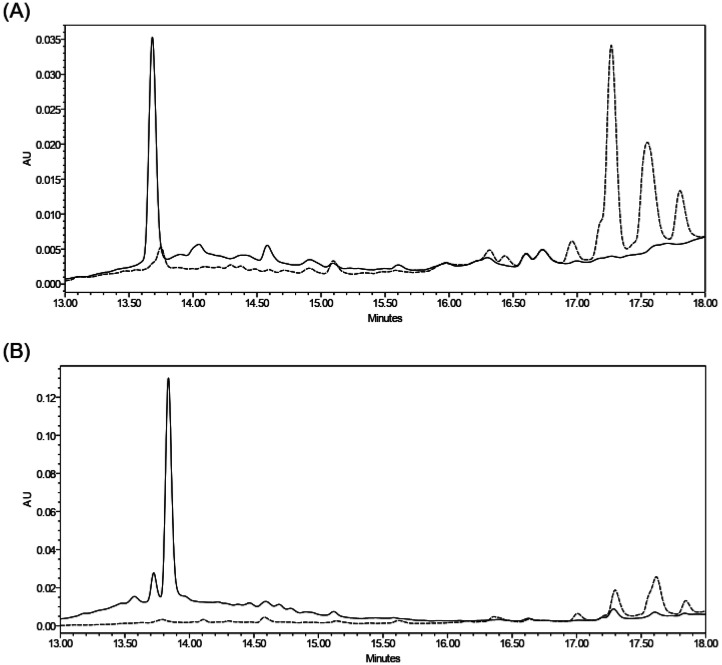
HPLC chromatogram of cell-free culture supernatant from *Lysobacter enzymogenes* B25. Chromatograms were obtained by injection of 10 µL of sample and detected by UV at 254 nm. (A) Potential metabolites produced by B25 strain after growing in TSB 1/10 medium (dotted line) compared with the chromatogram of the culture medium (solid line). (B) Different production of metabolites after growing in rich medium VEG (solid line) versus poor medium TSB 1/10 (dotted line).

By comparison of HPLC chromatograms of the different supernatants with the chromatograms obtained for the culture media, several peaks were detected around 17–18 min as potential metabolites produced by strain B25 (Figure 6). As previously described for *L. enzymogenes* C3, B25 produces a higher quantity of metabolites in nutritionally limited media, such as TSB 1/10 (Figure 6A), than in rich media, such as VEG (Figure 6B) [18].

By HPLC-HRMS analysis, two peaks with similar retention times were identified (Figure S4). The first one was a [M + H]^+^ ion with m/z of 513.2960 and a molecular formula of C_29_H_41_N_2_O_6_, which could correspond to the secondary metabolite HSAF. Another identified peak was its precursor 3-deOH-HSAF with a [M + H]^+^ ion of 497.3014 and a molecular formula of C_29_H_41_N_2_O_5_. Moreover, an unidentified compound coeluted with the HSAF peak. To attempt its identification, the two coeluted peaks were purified by preparative HPLC and analysed by ESI-TOF mass spectrometry (Table 2). The m/z values obtained from one of these peaks support its identification as HSAF. The values from the other peak confirm the presence of a compound with a molecular weight of 510 and the empirical formula of C_29_H_38_N_2_O_6_. According to the literature, the identity of this compound could be alteramide A or B; both compounds have the same molecular formula [53],[54]. Therefore, our results indicate that B25 produces three metabolites belonging to the polycyclic tetramate macrolactam family: HSAF, its precursor 3-deOH-HSAF and alteramide A/B.

**Table 2.** ESI (+) High Resolution data of peaks purified by preparative HPLC.

Peaks	Ions	(m/z)	Molecular Formula	Possible identity
Peak 1	[M + H]^+^	513.2952	C_29_H_41_N_2_O_6_	HSAF
	[M + Na]^+^	535.2780	C_29_H_40_N_2_O_6_Na	C_29_H_40_N_2_O_6_
	[M-H + 2Na]^+^	557.2598	C_29_H_39_N_2_O_6_Na_2_	
Peak 2	[M + H]^+^	511.2806	C_29_H_39_N_2_O_6_	Alteramide A/B
	[M + Na]^+^	533.2610	C_29_H_38_N_2_O_6_Na	C_29_H_38_N_2_O_6_
	[M-H + 2Na]^+^	555.2432	C_29_H_37_N_2_O_6_Na_2_	

### 3.4. Role of metabolites in nematicidal activity of L. enzymogenes B25

**Figure 7.**
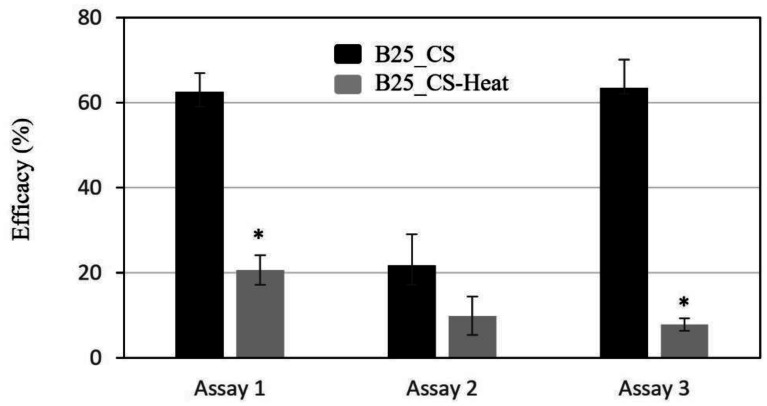
*In vitro* nematicidal activity of cell-free culture supernatant from *Lysobacter enzymogenes* B25. Results are shown as percentage of efficacy in inhibiting egg hatching of root knot nematodes for three independent experiments. B25 was grown in rich medium VEG (assays 1 and 2) and poor medium TSB 1/10 (assay 3). Within each trial, evaluated samples were cell-free culture supernatant (CS; black bars) and after heat treatment of this supernatant (CS-Heat; grey bars). Statistically different values (T-test; p < 0.05) are indicated by “*”.

Cell-free culture supernatants after growing B25 in both rich and poor media inhibit the hatching of RKN eggs *in vitro* (Figure 7). Similar efficacy results have been observed with samples obtained after growing in both media. Considering that the identified secondary metabolites are produced at higher concentrations in the poor medium than in the rich medium, our results suggest that other aspects are involved in the nematicidal activity.

To determine if the biocontrol activity of B25 against nematodes is due to the production of lytic enzymes, cell-free supernatants after the inactivation of enzymes by heat treatment were tested. The efficacy in egg hatching inhibition seems to be sensitive to high temperature; significant loss of activity was detected when the culture supernatant was heated at 80 °C for 15 min. To confirm that secondary metabolites are thermoresistant, HPLC analyses were conducted to compare the culture supernatants before and after treatment (Figure S5).

### 3.5. Induction of plant immune responses

**Figure 8.**
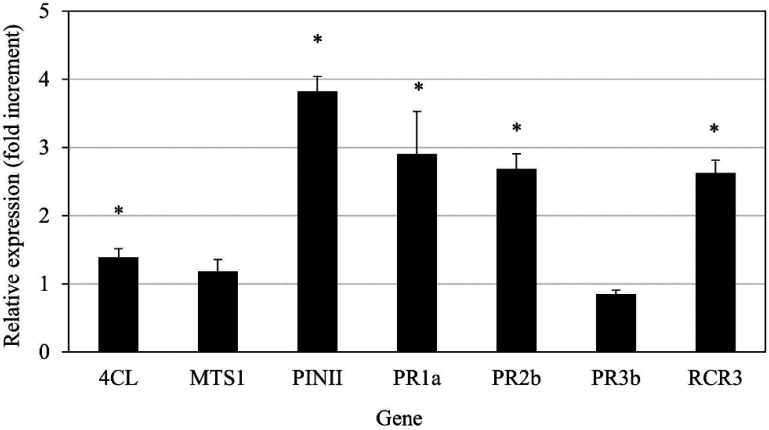
Relative gene expression levels of plant immune response marker genes in healthy tomato plants treated with *Lysobacter enzymogenes* B25. Results are shown as relative gene expression, normalized by the control plant expression values and the expression of Elongation factor 1α (EF1α). 4CL, 4-Coumarate:CoA ligase; MTS, Monoterpene synthase 1; PINII, Protease inhibitor II; PR1a, 2b and 3b, Pathogenesis-related 1a, 2b and 2b, respectively; RCR3, Required for *C. fulvum* resistance 3 (a papain-family cysteine protease). Statistically significant differences with the control plants (T-test; p < 0.05) are indicated by “*”.

The expression of several genes that are hallmarks of plant immune responses was tested in leaves after the application of *L. enzymogenes* B25 to the roots of healthy tomato plants. The application of B25 to the roots resulted in a mild overexpression (1.4–3.8 ×) of 5 out of the 7 genes tested in leaves (Figure 8), in the absence of nematodes.

## 4. Discussion

*Lysobacter enzymogenes* has been widely reported as a biocontrol agent of fungal pathogens [10],[25],[55]–[57]. However, few reports have been published focusing on the nematicidal activity of *L. enzymogenes*, particularly with *L. enzymogenes* C3 [13],[14]. In fact, only one of these studies report the nematicidal activity in plants under growth chamber conditions. Yuen and coworkers published the activity of strain C3 controlling the infestation of cyst nematodes, *H. schachtii* and *H. glycines*, in sugarbeet, cabbage, and soybean.

In the present study, the efficacy of *L. enzymogenes* B25 controlling different species of root-knot nematodes, such as *M. incognita, M. javanica*, and *M. hapla*, in tomato plants is shown. In preliminary experiments under *in vitro* conditions, B25 showed a direct effect on eggs and juveniles in the mixed population of *M. incognita* and *M. javanica* (Viladecans) and *M. javanica* (Almería) reaching 80–90 % of control depending on the population and the stage of the nematode. In tomato plants, B25 controls RKN infestation with efficacies of 50–95 % depending on the population and the pathogen pressure. In the three bioassays described in this work under growth chamber conditions, the number of nematodes per 100 g of soil applied as initial inoculum was 10 times higher (bioassay 1) or 16 times higher (bioassay 2–3) than that recommended as a threshold to initiate nematicide treatments to control *Meloidogyne* in tomato [58]. Therefore, we can ensure that the level of inoculum to which the plants were subjected in the three bioassays would correspond to a field with a high infestation level. Despite having inoculated bioassays 2 and 3 with more nematodes, it was in bioassay 1 where the number of nematodes per g of the root was 3.5 times higher, once again highlighting the high rate of reproduction of the Viladecans population. In this case (bioassay 1), the efficiencies reached by B25 were significantly lower than in the chemical control. Under lower pathogen pressure conditions than in the first bioassay, the results achieved by B25 were equal to or even significantly higher than the efficacy achieved by the systemic nematicide oxamyl.

As it is demonstrated in this study, one aspect linked to the biological control of *L. enzymogenes* B25 is its capacity to colonize and infect the plant-parasitic-nematodes. B25 shows the ability to form biofilms which play an important role in the colonization of surfaces (soil, roots, or shoots of plants) and enable proliferation in the desired niche. Also, microorganisms form biofilms as a strategy to overcome stress, such as nutrient depletion or changes in pH [59]. In addition, B25 can reach surfaces by swarming and twitching motility. These migration mechanisms allow it to search for nutrients, interact with its surroundings, survive under adverse environmental conditions and move toward the nematodes. It is to be noted that swarming motility is operationally defined as a rapid multicellular bacterial surface movement powered by rotating flagella. Although B25 does not have flagella, it shows swarming motility thanks to the production of surfactants that reduce the tension between bacterial cells and surfaces. In addition, external cell appendages, such as T4P, can also be involved in swarming motility [60]. Twitching motility is associated with the extension and retraction of T4P, and it has been widely reported in *L. enzymogenes* [61]–[64]. However, this is the first report describing that *L. enzymogenes* increases its twitching motility in the presence of *M. incognita*, suggesting that more bacterial cells might be trying to move toward the nematode. Similar behavior has also been described when *L. enzymogenes* were co-cultured with the oomycete *Pythium aphanidermatum* [10]. In addition to the active propagation or biofilm formation presented in the present study, the persistence of B25 in the soil and whether it can colonize plant tissues has been assessed recently [65]. The study showed that the persistence of B25 in the tomato root/rhizosphere was greater than 28 days and that B25 applied to the rhizosphere was also detected in the shoots, which shows that this strain is able to colonize plant tissues.

Moreover, *L. enzymogenes* B25 produces and secretes a large number of enzymes related to biocontrol, particularly with nematicidal activity. For instance, as chitin is the main component of the eggshell and cuticle of nematodes, chitinases from B25 could affect nematodes via eggshell digestion and cuticle hydrolysis [13],[66]. Other extracellular enzymes secreted by B25 are proteases, including gelatinases. They degrade denatured collagen, the main constitutive component of nematode cuticle [67],[68]. For example, in a previous study, gelatinases produced by *Lysobacter capsici* YS1215 have been reported to degrade the cuticle, inducing mortality, in juvenile nematodes [69],[70]. B25 also secretes trypsin, a serine protease that exhibits nematicidal effects by destroying the hatched eggs of *M. incognita* [71]–[73]. Other hydrolytic enzymes produced by B25 are lipases and cellulases. Lipases are enzymes that hydrolyze glycerol esters, preferably long-chain fatty acids. The lipid content (including reserves) of free-living nematodes and plant parasitic nematodes ranges from 11 to 67% of dry weight. The nematicidal activity of lipases has been reported in *Tylenchorhynchus dubius*, where lipases reduced the population by up to 100%. In addition, bacterial lipases belonging to the families *Microbacteriaceae, Xanthomonadaceae, Enterobacteriaceae, Burkholderiaceae* and *Pseudomonadaceae* have also been shown to control *Bursaphelencus xylophilus* populations [74]. Also, the strong lipase activity of *Bacillus thuringiensis, Bacillus megaterium* and *Bacillus amyloliquefaciens* causes mortality to *Xiphinema index* nematode [75]. Furthermore, lipase C14 produced by B25 might affect the vitellin and lipid layers on the egg cover of invertebrates and esterase lipase (C8) might hydrolyze some nematode egg surface components (short-chain esters) and facilitate the access of some metabolites released by the bacterium with nematicidal activity [76].

In addition to extracellular enzymes, B25 is able to produce several metabolites belonging to the polycyclic tetramate macrolactam family, i.e. HSAF, 3-deOH-HSAF, and alteramide A/B. Numerous reports have described the antifungal activity of these secondary metabolites and their role to control plant disease [18],[19],[53],[77]. On the contrary, few reports related to the involvement of these metabolites in the activity of *L. enzymogenes* against nematodes have been published. In addition to our study, the role of HSAF in the biocontrol of plant-parasitic nematodes has been assessed recently [14]. By testing the nematicidal activity of a mutant strain blocked in HSAF production, it was shown that the production of this metabolite is an important mechanism involved in the biocontrol activity against cyst nematodes. However, our results indicate that the contribution of secondary metabolites produced by B25 in inhibiting root-knot nematodes egg hatching is minor compared to the role of lytic extracellular enzymes. According to previous studies on *L. enzymogenes* C3, B25 produces a higher quantity of HSAF under nutrient-poor conditions [18]. In a rich medium, cellular c-di-GMP levels increase and form a complex with Clp, blocking the activation of the HSAF biosynthesis operon [16]. Another study has also associated increasing intracellular c-di-GMP levels with PilR, a response regulator that controls T4P synthesis and twitching motility [63]. Thus, PilR activates twitching motility while HSAF production is downregulated. This fact is consistent with the increased twitching motility of B25 in the presence of nematodes nearby and a negligible role of the secondary metabolites, probably due to decreased production.

Finally, the biocontrol activity of B25 could be due in part –the extent of defense gene overexpression is mild– to the enhancement of plant immunity at the systemic level (e.g. induced systemic response and systemic acquired resistance). This phenomenon was previously reported for *L. enzymogenes* C3 in tall fescue (*Festuca arundinacea*) [26]. The authors of this study showed that peroxidases are involved in the induction of plant resistance to fungal pathogens by *L. enzymogenes*, but the exact role of the enzymes in that process remains unclear.

## 5. Conclusions

In this study, a novel strain of *L. enzymogenes* has been identified as a biocontrol agent against *Meloidogyne* spp. and its mode of action has been studied. B25 appears to employ twitching motility to draw near plant-parasitic nematodes, colonizing and invading them. Then, upon contact with the nematodes, B25 kills them by deploying a set of extracellular lytic enzymes that are capable of digesting the main chemical constituents of the nematode cuticle and eggshells (protein, chitin, and lipids), conferring its nematicidal activity. In addition, the induction of resistance in the host plants by B25 may also contribute to its biocontrol activity. Although B25 produces polycyclic tetramate macrolactam metabolites, such as HSAF, our results suggest a minor contribution of these metabolites in the control of RKN infestation.

The good efficacy of *L. enzymogenes* B25 controlling RKN infection in tomato plants has been demonstrated under growth chamber and greenhouse conditions. Moreover, several field trials assessing B25 against RKN (*Meloidogyne* spp.) and potato cyst nematodes (*G. pallida* and *G. rostochiensis*) have confirmed its efficacy (additional experiments have not been published yet). All these results highlight the potential of *L. enzymogenes* B25 to control plant parasitic nematodes as an environmentally friendly method.
