# Supplementary material for: Characterization of Lysobacter enzymogenes B25, a potential biological control agent of plant-parasitic nematodes, and its mode of action
Source: AIMS Microbiol. 2023 Mar 1;9(1):151–76. doi: 10.3934/microbiol.2023010 (PMC9988411; doi:10.3934/microbiol.2023010)
Supplement: Supplementary file 1 [file microbiol-09-01-010-s001.pdf]

Microbiology

AIMS Microbiology, 9(1): 151–176.
DOI: 10.3934/microbiol.2023010
Received: 09 December 2022
Revised: 16 February 2023
Accepted: 22 February 2023
Published: 01 March 2023

http://www.aimspress.com/journal/microbiology

*Research article*

# Characterization of *Lysobacter enzymogenes* B25, a potential biological control agent of plant-parasitic nematodes, and its mode of action

**Sònia Martínez-Servat*, Lola Pinyol-Escala, Oriol Daura-Pich, Marta Almazán, Iker Hernández, Belén López-García*, Carolina Fernández**

Futureco Bioscience, S.A, Olèrdola, Barcelona, Spain

* **Correspondence:** sonia@futurecobioscience.com; belen@futurecobioscience.com; Tel: + (34)938182891

## Supplemental

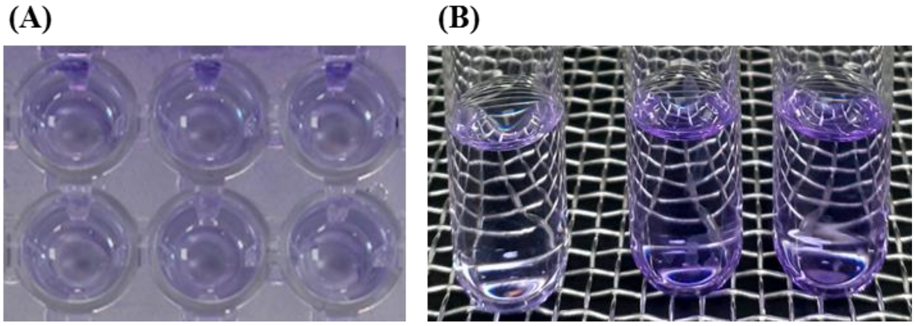


**Figure S1.** *Lysobacter enzymogenes* B25 biofilm formation on different surfaces: (A) polystyrene plate and (B) glass tubes. After B25 was grown in wells and tubes, they were stained with crystal violet 0.1 % and filled with ethanol. Violet color indicates cell adhesion to the surface and biofilm formation.

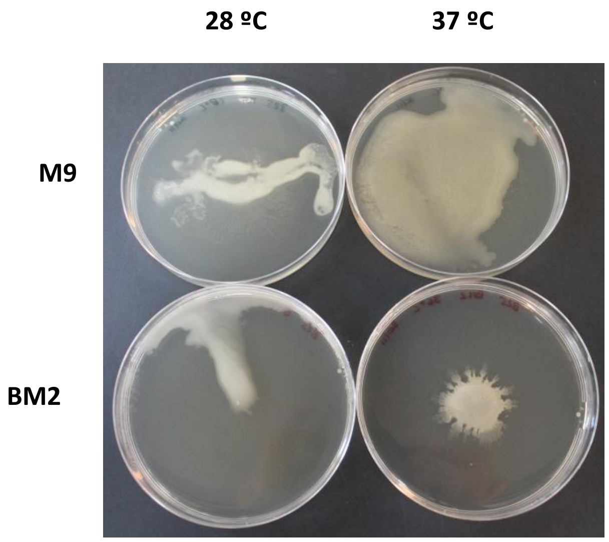


**Figure S2.** Swarming motility of B25 was measured on modified M9 medium and BM2 medium solidified with 0.5% Noble agar after 24 hours incubation at 28 ℃ or 37 ℃.

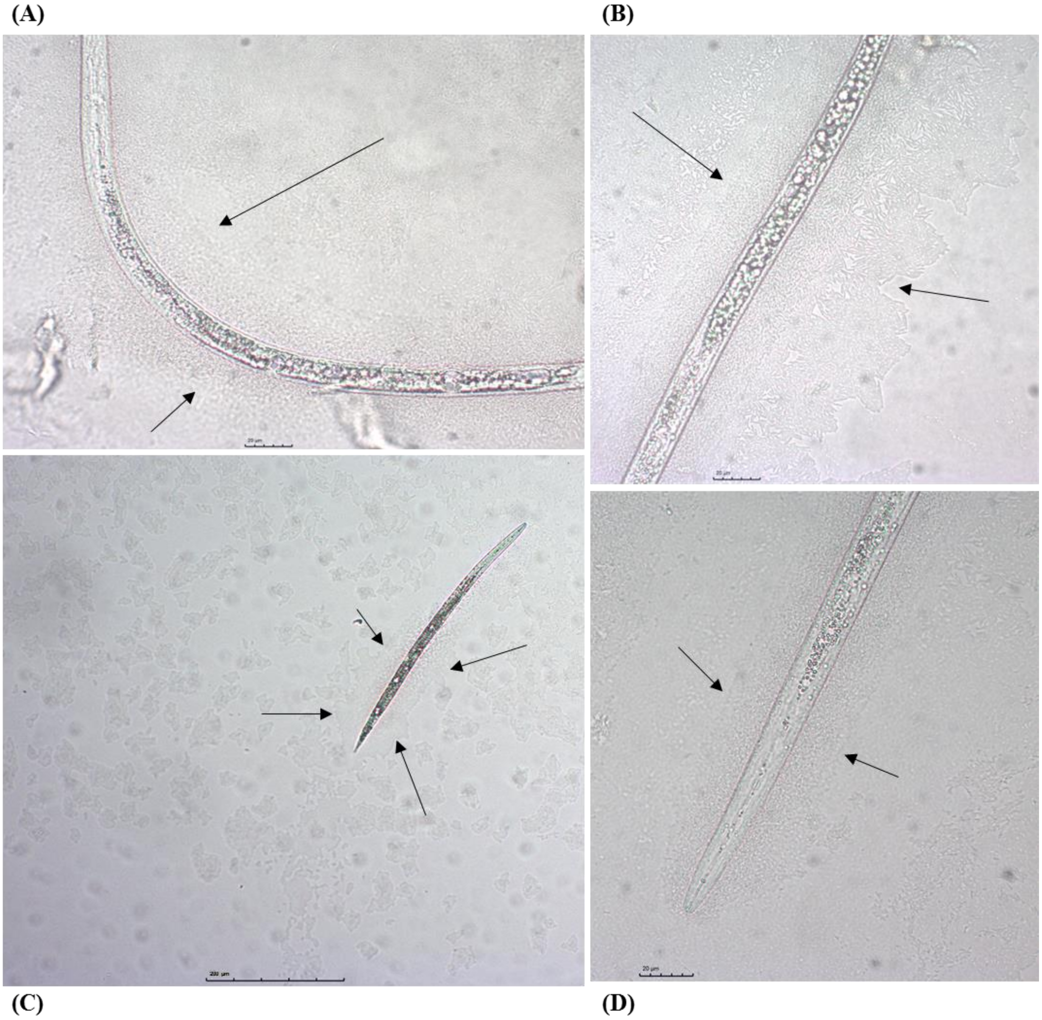


**Figure S3.** Twitching motility of B25 in the presence *of Meloidogyne incognita*. (A, B and D) Magnification x400. (C) Magnification x100. Arrows indicate the high-density cell envelope around the *M. incognita.*

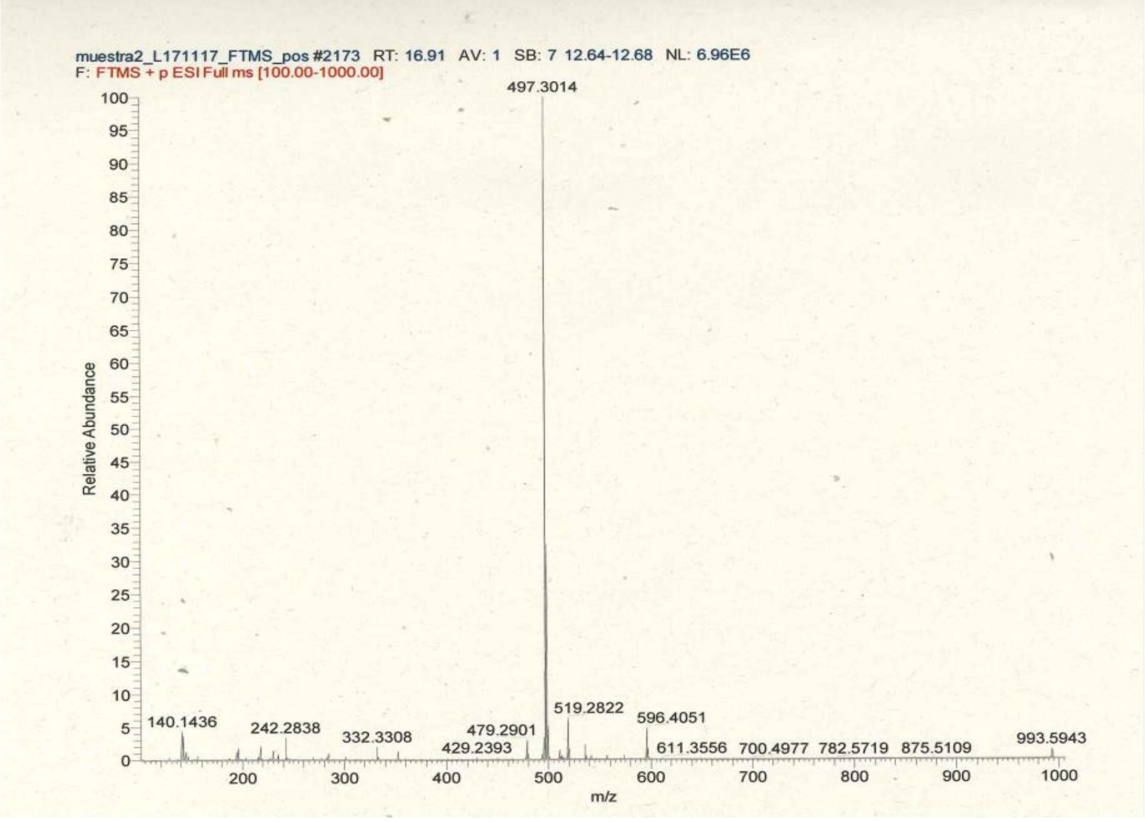


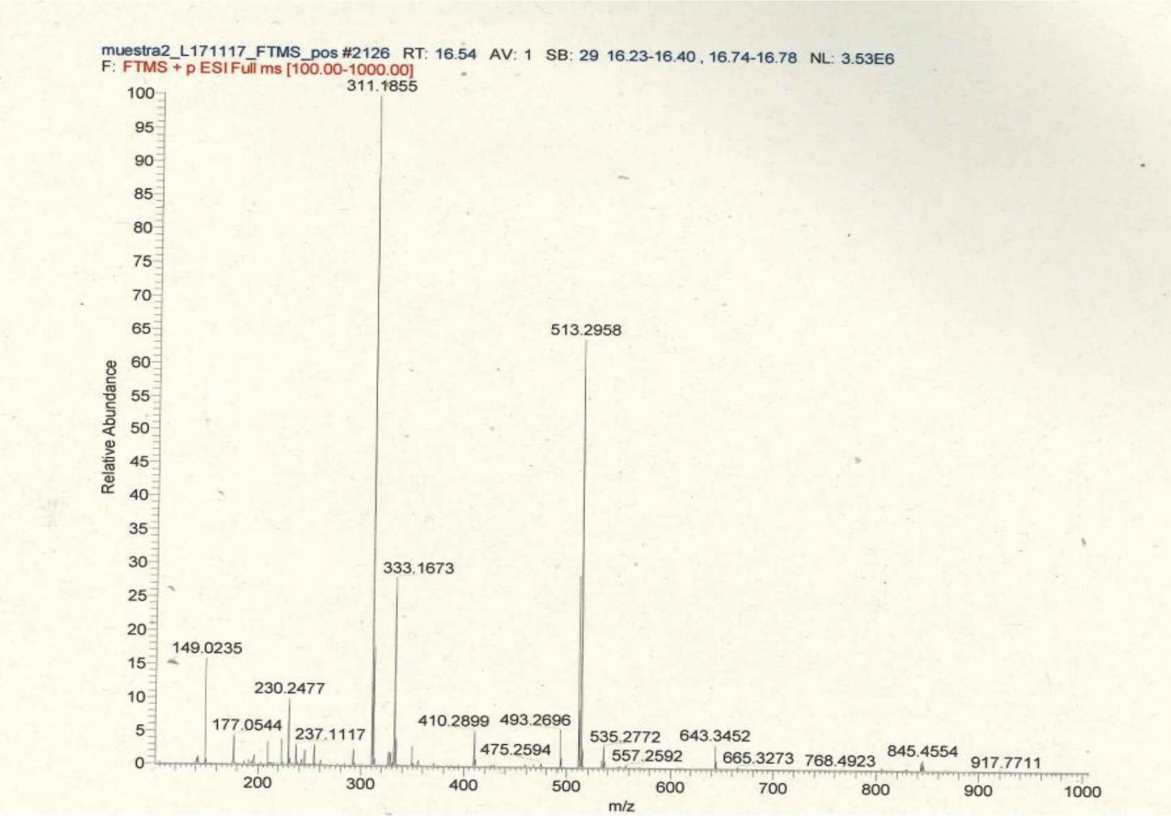


**Figure S4:** FTMS spectrum of identified peaks by HPLC-HRMS analysis.

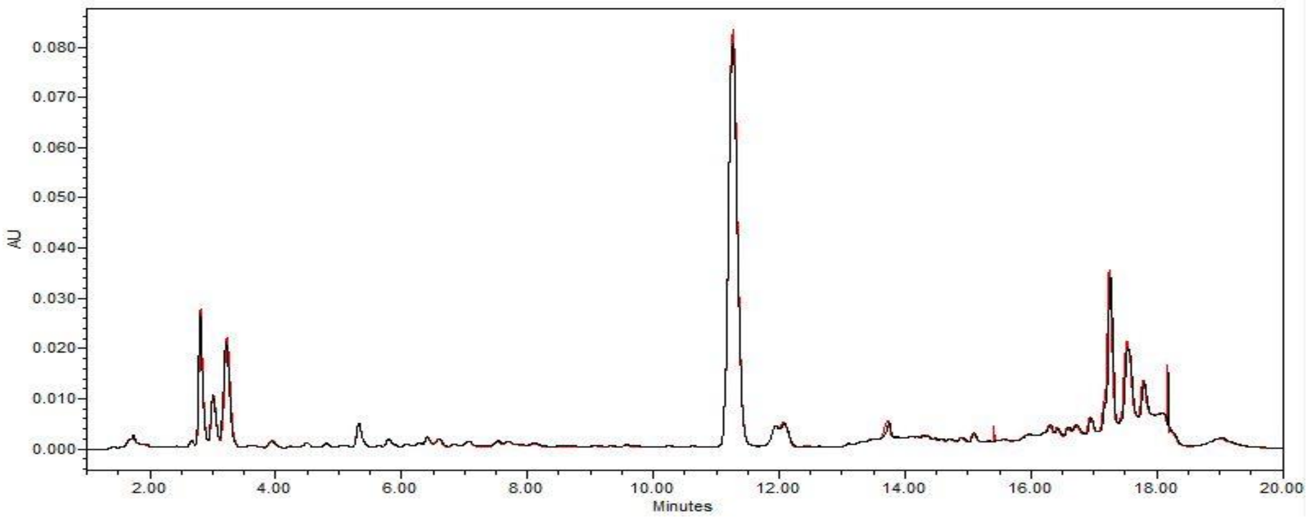


**Figure S5.** HPLC chromatograms of cell-free culture supernatant from B25 after growing in TSB 1/10 medium (black) and after heat treatment at 80 °C for 15 min (red). Chromatograms were obtained by injection of 10 µL of sample and detected by UV at 254 nm.

**Table S1.** Results of 4 greenhouse assays performed at Futureco Bioscience S.A. facilities on tomato cv. Durinta. Strain B25 was applied 4 times in 7–14 days intervals as described in Materials and Methods section.

| Trial code | Target | Dose (CFU's/mL) | Pathogen pressure in control plants Eggs/g of root | % Efficacy of B25 Eggs/g of root | % Efficacy chemical reference | % Efficacy biological reference 2 |
|---|---|---|---|---|---|---|
| 160314 IN MT | *Meloidogyne javanica* + *Meloidogyne incognita* | 3,03E + 08 | 4239 | 46.98% | (Oxamyl 10%) 96.98% | - |
| 160307 IN MT | *Meloidogyne javanica* + *Meloidogyne incognita* | 2,67E + 08 | 5828 | 60.96% | (Oxamyl 10%) 98.25% | - |
| 181211 IN MT | *Meloidogyne hapla* | 1,74E + 08 | 2274 | 90.09% | (Flupyram 40%) 85.49% | (*Bacillus firmus* 5%) 6.19% |
| 190122 IN MT | *Meloidogyne hapla* | 1,05E + 08 | 4138 | 94.59% | (Flupyram 40%) 81.56% | (*Bacillus firmus* 5%) 0% |

**Table S2.** Primers used in this study.

| Gene | Genbank Accession | Cultivar | Primers (forward/reverse; 5'-3') | Product size (nt)[a] | E |
|---|---|---|---|---|---|
| PR1a | DQ159948 | Jin Bao/ Castlemart | CCAAGCTATAACTACGCTACCAAC<br>GCAAGAAATGAACCACCATCC | 139 | 1.981 |
| PR3b | Z15140 | Jin Bao/ Castlemart | AACTATGGGCCATGTGGAAGA<br>GGCTTTGGGGATTGAGGAG | 128 | 2.043 |
| RCR3 | NM_001320315.1 | Money Maker | TACAAGCCGTAACTAAACAG<br>TCATATACCCATTCTCACCC | 209 | 2.053 |
| PINII | X94946 | Jin Bao/ Castlemart | AATTATCCATCATGGCTGTTCAC<br>CCTTTTTGGATCAGATTCTCCTT | 254 | 1.997 |

*Continued on next page*

| Gene | Genbank Accession | Cultivar | Primers (forward/reverse; 5'-3') | Product size (nt)[a] | E |
|---|---|---|---|---|---|
| PR2b | LOC543986 | Jin Bao | GGACACCCTTCCGCTACTCTT<br>TGTTCCTGCCCCTCCTTTC | 81 | 2.052 |
| MTS1 | AY840091 | Money Maker<br>/Castlemart | TTTGGGGACATCTTCGGATGAA<br>CTACTCGAGTTACTTGAGAGCGAATGCAAC | 309 | 2.042 |
| 4CL | XM_004235822 | Pausa | ACACACAAAGGCTTAGTCACGA<br>AACAGAGGCAACACACACATCA | 103 | 1.999 |
| EF1α | XM_004240531 | M82/<br>Heinz 1706 | GATTGGTGGTATTGGAACTGTC<br>AGCTTCGTGGTGCATCTC | 129 | 1.977 |

[a]Product sizes may vary among tomato cultivars.

**Table S3.** Raw Ct values of qPCR analysis.

| Target_Pos | Target_treat | Target_Cp | Target | Ref_Pos | Ref_Treat | Ref_Cp | Ref |
|---|---|---|---|---|---|---|---|
| A1 | T1 | 23,77 | 4CL | A9 | T1 | 21,67 | EF1a |
| B1 | T1 | 23,34 | 4CL | B9 | T1 | 21,62 | EF1a |
| C1 | T1 | 23,52 | 4CL | C9 | T1 | 21,67 | EF1a |
| B2 | T4 | 22,73 | 4CL | B10 | T4 | 21,4 | EF1a |
| C2 | T4 | 22,75 | 4CL | C10 | T4 | 21,6 | EF1a |
| D2 | T4 | 22,9 | 4CL | D10 | T4 | 21,48 | EF1a |
| A9 | T1 | 21,94 | MTS1 | A9 | T1 | 21,67 | EF1a |
| B9 | T1 | 21,96 | MTS1 | B9 | T1 | 21,62 | EF1a |
| C9 | T1 | 21,82 | MTS1 | C9 | T1 | 21,67 | EF1a |
| B10 | T4 | 21,45 | MTS1 | B10 | T4 | 21,4 | EF1a |
| C10 | T4 | 21,7 | MTS1 | C10 | T4 | 21,6 | EF1a |
| D10 | T4 | 21,42 | MTS1 | D10 | T4 | 21,48 | EF1a |
| A5 | T1 | 17,96 | PINII | A9 | T1 | 21,67 | EF1a |
| B5 | T1 | 18 | PINII | B9 | T1 | 21,62 | EF1a |
| C5 | T1 | 18,36 | PINII | C9 | T1 | 21,67 | EF1a |
| B6 | T4 | 16,15 | PINII | B10 | T4 | 21,4 | EF1a |
| C6 | T4 | 16,14 | PINII | C10 | T4 | 21,6 | EF1a |
| D6 | T4 | 15,95 | PINII | D10 | T4 | 21,48 | EF1a |
| A1 | T1 | 16,72 | PR1a | A9 | T1 | 21,67 | EF1a |
| B1 | T1 | 16,44 | PR1a | B9 | T1 | 21,62 | EF1a |
| C1 | T1 | 16,52 | PR1a | C9 | T1 | 21,67 | EF1a |
| B2 | T4 | 14,7 | PR1a | B10 | T4 | 21,4 | EF1a |
| C2 | T4 | 14,79 | PR1a | C10 | T4 | 21,6 | EF1a |
| D2 | T4 | 14,64 | PR1a | D10 | T4 | 21,48 | EF1a |
| A5 | T1 | 24,68 | PR2b | A1 | T1 | 21,77 | EF1a_PR2 |
| B5 | T1 | 24,29 | PR2b | B1 | T1 | 21,5 | EF1a_PR2 |
| C5 | T1 | 25,44 | PR2b | C1 | T1 | 14,97 | EF1a_PR2 |
| B6 | T4 | 22,64 | PR2b | B2 | T4 | 15,03 | EF1a_PR2 |
| C6 | T4 | 22,96 | PR2b | C2 | T4 | 14,55 | EF1a_PR2 |
| D6 | T4 | 22,64 | PR2b | D2 | T4 | 14,64 | EF1a_PR2 |
| A5 | T1 | 19,56 | PR3b | A9 | T1 | 21,67 | EF1a |
| B5 | T1 | 19,59 | PR3b | B9 | T1 | 21,62 | EF1a |
| C5 | T1 | 19,61 | PR3b | C9 | T1 | 21,67 | EF1a |
| B6 | T4 | 19,65 | PR3b | B10 | T4 | 21,4 | EF1a |
| C6 | T4 | 19,67 | PR3b | C10 | T4 | 21,6 | EF1a |
| D6 | T4 | 19,62 | PR3b | D10 | T4 | 21,48 | EF1a |
| A1 | T1 | 26,69 | RCR3 | A9 | T1 | 21,67 | EF1a |
| B1 | T1 | 26,63 | RCR3 | B9 | T1 | 21,62 | EF1a |
| C1 | T1 | 26,59 | RCR3 | C9 | T1 | 21,67 | EF1a |
| B2 | T4 | 24,67 | RCR3 | B10 | T4 | 21,4 | EF1a |

*Continued on next page*

| Target_Pos | Target_treat | Target_Cp | Target | Ref_Pos | Ref_Treat | Ref_Cp | Ref |
|---|---|---|---|---|---|---|---|
| C2 | T4 | 24,71 | RCR3 | C10 | T4 | 21,6 | EF1a |
| D2 | T4 | 24,88 | RCR3 | D10 | T4 | 21,48 | EF1a |

The Cp of NTCs (no-template controls) was in all cases > 30

PR2 required different qPCR conditions so the reference gene was analyzed under the same conditions

AIMS Press

© 2023 the Author(s), licensee AIMS Press. This is an open access article distributed under the terms of the Creative Commons Attribution License (http://creativecommons.org/licenses/by/4.0)
